# Morphology Control of Polymer–Inorganic Hybrid Nanomaterials Prepared in Miniemulsion: From Solid Particles to Capsules

**DOI:** 10.3390/polym16212997

**Published:** 2024-10-25

**Authors:** Olaia Álvarez-Bermúdez, Inés Adam-Cervera, Katharina Landfester, Rafael Muñoz-Espí

**Affiliations:** 1 Institute of Materials Science (ICMUV), Universitat de València, c/Catedràtic José Beltrán 2, 46980 Paterna, Spain; 2 Max Planck Institute for Polymer Research, Ackermannweg 10, 55128 Mainz, Germany

**Keywords:** organic–inorganic, hybrid, nanoparticle, nanocapsule, miniemulsion, morphology control

## Abstract

The preparation of so-called hybrid nanomaterials has been widely developed in terms of functional and morphological complexity. However, the specific control of the arrangement of organic and inorganic species, which determines the properties of the final material, still remains a challenge. This article offers a review of the strategies that have been used for the preparation of polymer–inorganic hybrid nanoparticles and nanocapsules via processes involving miniemulsions. Different polymer–inorganic nanostructures are classified into four main groups according to the sequential order followed between the synthesis of the polymer and the inorganic species, and the presence or not of their counterpart precursors. The minimization of the energy of the system governs the self-assembly of the different material components and can be addressed by the miniemulsion formulation to reduce the interfacial tensions between the phases involved. The state of the art in the preparation of hybrid nanoparticles is reviewed, offering insight into the structural possibilities allowed by miniemulsion as a versatile synthetic technique.

## 1. Introduction

Polymer–inorganic hybrid materials are the result of the synergistic combination of inorganic functionalities (e.g., catalytic activity, magnetism, or luminescence) with the biocompatibility, high stability, and easy processability features of polymers. Furthermore, some inorganic reinforcements contribute to the mechanical, thermal, chiral, and electric properties of specific polymer matrices. Therefore, the resulting properties of hybrids go beyond the simple addition of the individual properties of the forming components. The morphology of this type of material plays an essential role in the final properties, and its relevance increases with complexity and the reduction in the size of the system. However, from a synthetic point of view, the achievement of hybrid nanomaterials with precisely controlled structure still remains the “holy grail” looked for by many researchers in materials science.

In general, polymer particles can be prepared by many different methods, summarized and compared in Table 1 according to their respective advantages and disadvantages. Some of the methods included in this table imply polymerization, while in others the polymer is already preformed, and the particles are prepared following, for instance, precipitation or spraying steps. Among these techniques, not all are equally suited for either obtaining nanosized materials or integrating and encapsulating inorganic components. In this respect, miniemulsion polymerization is quite special because it offers high versatility and allows for the incorporation of inorganic functionalities at different locations within a polymer matrix; it stands out in colloidal chemistry for the preparation of polymer–inorganic hybrid nanoparticles, overcoming the structural limitations of other methods [1]. For instance, layer-by-layer deposition, suspension, or conventional emulsion polymerization processes are commonly restricted to the production of hybrid nanostructures with inorganic particles located on the polymer surface or of single inorganic particles covered by a polymer shell.

In a previous work [44], we described the theory and the state of the art in the preparation of nanoparticles of any nature in miniemulsion. Herein, we focus on the advances on the different types of polymer–inorganic hybrid nanoparticles prepared in miniemulsion and the synthetic strategies used to control their structure. Besides “standard” miniemulsion polymerization in the presence of organic surfactants, we will also review other techniques involving miniemulsions, such as Pickering miniemulsion polymerization or miniemulsion–solvent evaporation.

Miniemulsions are kinetically stabilized emulsions of two immiscible liquids with a droplet size between 50 and 500 nm, which typically require high-shear forces (e.g., high-power ultrasounds or high-pressure homogenization) for being homogenized and the presence of an osmotic pressure agent inside of the droplets. Depending on the polar nature of the phases, we differentiate between direct miniemulsions, when a non-polar liquid is dispersed within a polar continuous phase (typically oil-in-water, O/W, although other polar phases are also possible), and inverse miniemulsions, in the opposite case (typically water-in-oil, W/O). In both systems, the nanodroplets provide confined spaces in which chemical reactions can take place. For this reason, assuming ideally that no coalescence or interdroplet diffusion occurs, miniemulsions are considered dispersions of “nanoreactors”. Therefore, miniemulsions have been extensively exploited for the synthesis of polymer [11,45], inorganic [46,47], and organic–inorganic hybrid [1,48,49] nanoparticles and nanocapsules.

Regarding the nomenclature, it is necessary to clarify the terms *miniemulsion* and *nanoemulsion*, which appear simultaneously in the literature. *Miniemulsion* is a more general term, whereas *nanoemulsion* denotes a miniemulsion with droplet size below 100 nm. To avoid any confusion, one should also point out that, in contrast, *microemulsion* is a different concept that refers to thermodynamically stable systems, whereas miniemulsion and nanoemulsions are thermodynamically unstable.

**Figure 1:**
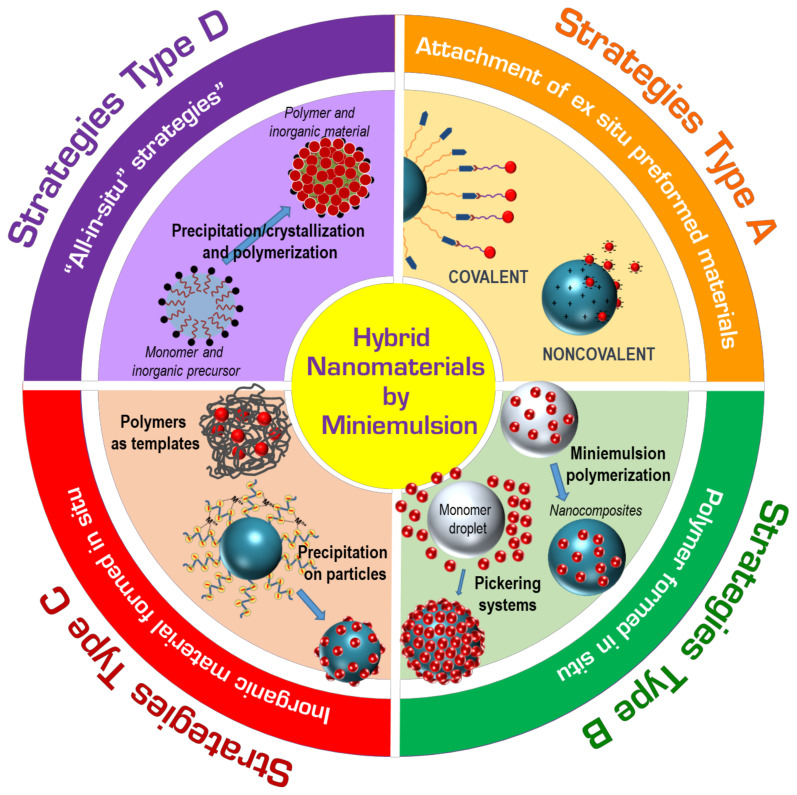
General scheme of the synthetic strategies for preparation of polymer–inorganic hybrid nanomaterials prepared in miniemulsion.

This work considers those cases in which at least one of the steps of the preparation takes place in miniemulsion. According to the presence or the absence of the counterpart species or precursors during the synthesis of the polymer and/or the inorganic components, we propose a classification of the different strategies based on the groups listed below and represented schematically in Figure 1.

- **Strategies type A** (both polymer and inorganic components preformed ex situ): they consist of the self-assembly of preformed polymer and inorganic components and involve typical processes of inorganic deposition on functionalized polymers, solvent evaporation, or heterocoagulation techniques.
- **Strategies type B** (polymer formed in situ): they involve polymerization processes in the presence of preformed inorganic components. This group includes typical miniemulsion polymerization, interfacial polymerization, semi-batch polymerization, seed emulsion polymerization in the presence of inorganic components synthesized ex situ, or Pickering emulsion.
- **Strategies type C** (inorganic material formed in situ)**:** they use the polymer nanoparticles as supports for inorganic synthesis. In these processes, the in-situ formation of the inorganic species takes place on the preformed polymer by inorganic crystallization, precipitation/mineralization, or interfacial sol–gel processes.
- **Strategies type D** (both polymer and inorganic components formed in situ): they are “all-in-situ” strategies are challenging syntheses related to the formation of the inorganic nanoparticles via hydrolysis and condensation reactions of inorganic precursors occurring simultaneously to miniemulsion polymerization processes.
- Finally, **multistep strategies** involve complex cases resulting from the mixture of pure synthetic processes, which occur either simultaneously or consecutively.

We have divided this review into “solid nanoparticles” (Section 2) and “nanocapsules” (Section 3), being aware that this differentiation between particles and capsules may be sometimes ambiguous and almost arbitrary. In this regard, our criterion is the following: we consider “capsules” as hollow spheres formed by a polymer, inorganic, or hybrid shell that encapsulates a core of an active or functional liquid component. In contrast, solid systems containing (i.e., “encapsulating”) an active/functional component are considered “particles”. The literature is sometimes confusing, because solid particles can “encapsulate”—and this is actually the term often used—active principles, but they are not “capsules” according to our nomenclature. In a simplified way, we speak about “solid particles” when all components are solid, while we can refer to “capsules” when there is a core–shell morphology with a liquid core. This classification is mainly for didactic purposes, taking into account our focus on morphology control. Indeed, so-called “nanocapsules” are nothing else but a certain morphology of “nanoparticles”.

## 2. Preparation of Hybrid Solid Nanoparticles

### 2.1. Basic Principles for Structure Control in Hybrid Nanoparticles

The specific arrangement of the different components in polymer–inorganic hybrid nanostructures synthesized in miniemulsion results from self-assembly processes driven by the minimization of the global energy of the system (*E*).

(1) $$E=\sum_{\mathrm{ij}}A_{\mathrm{ij}}\gamma_{ij}=A_{\mathrm{PW}}\gamma_{\mathrm{PW}}+A_{\mathrm{IW}}\gamma_{\mathrm{IW}}+A_{\mathrm{IP}}\gamma_{IP}$$

**Figure 2:**
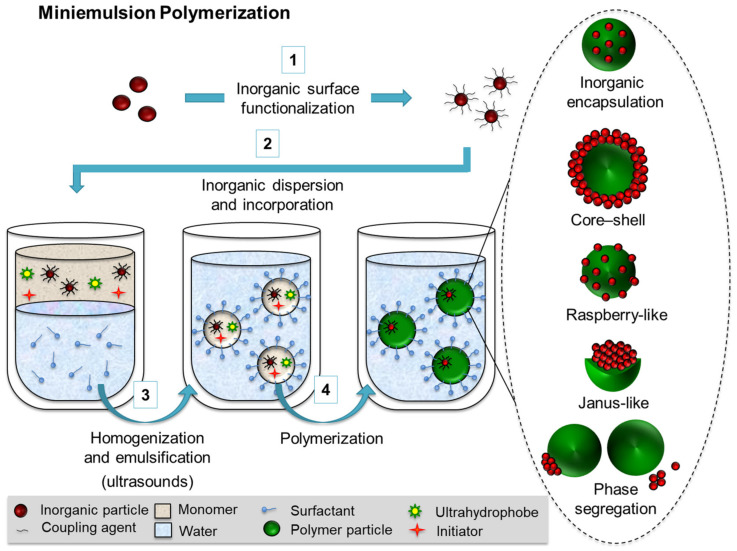
Scheme of the preparation of polymer–inorganic nanoparticles by miniemulsion polymerization and the resulting hybrid morphologies.

For an aqueous dispersion of hybrid nanoparticles, *E* is expressed according to Equation (1) as the sum of the individual interfacial energies (*E_i_*) between the different phases (*i*,*j*), that is, the polymer (*P*), the inorganic species (*I*), and the aqueous phase (*W*). In the equilibrium, the development of the preferred hybrid morphology takes place by lowering the interfacial areas (*A_ij_*) and interfacial tensions (*γ_ij_*) related to the three phases. According to this theory, the control of the inner structure of hybrid nanoparticles synthesized in miniemulsion can be addressed by tuning the energy balance of the system. González-Ortiz and Asua [50] proposed a mathematical correlation between the interfacial tensions (*γ*_PW_, *γ*_IP_, and *γ*_IW_) and the development of different hybrid structures. The authors established morphology diagrams that were used to evaluate the theoretical predictions in comparison to the empirical results reported for different polymer–inorganic combinations [50,51]. The study points out the nature and the amount of the surfactant [52], the type of initiation [53] of the polymerization, and the differences of polarity of the monomer/polymer [54] versus the inorganic species as the three main parameters influencing the interfacial tensions in a miniemulsion polymerization process. Figure 2 schematically presents the synthesis of polymer–inorganic nanoparticles by miniemulsion polymerization and the different achievable morphologies. The miniemulsion formulation strongly determines the homogeneous distribution of the inorganic species within the polymer matrix, the phase segregation of the species with the formation of Janus-like or core–shell morphologies, or the complete separation of the polymer and the inorganic materials.

The concentration of the surfactant determines the interfacial tension between the polymer (*γ*_PW_) and the inorganic components (*γ*_IW_) with the aqueous phase. At low surfactant concentrations, the phase with the lower interfacial tension with respect to water will be exposed at the droplet interface. The use of high surfactant concentrations lowers both *γ*_PW_ and *γ*_IW_, whose difference becomes negligible. Then, the development of the hybrid morphology relies on the interfacial tension between the polymer and the inorganic components (*γ*_PI_). According to these considerations, inorganic encapsulation is favored at low concentrations of surfactant, whereas high concentrations allow for phase segregation and Janus-like structures. In the case of a free-radical polymerization process in direct miniemulsion, the initiator has a direct influence on the interfacial tension between the polymer and the aqueous phase (*γ*_PW_). Water-soluble initiators, such as potassium peroxydisulfate (KPS), commonly contain charged groups. Therefore, the radicals generated in the aqueous phase enter the hydrophobic droplets and lead to an increase in the charge at the droplet interface. Consequently, the decrease in the interfacial tension, *γ*_PW_, favors a redistribution of interfacial areas, leading to an increase in the interfacial area between the hybrid nanoparticle and water (*A*_WP_). This process supports the encapsulation of hydrophobic inorganic species within the polymer matrix, minimizing their exposure to the aqueous phase. When oil-soluble initiators, such as 2,2′-azobis(2-methylpropionitrile) (AIBN) or 2,2′-azobis-(2-methylbutyronitrile) (AMBN), are used, the generation of the radicals takes place within the droplet confinement, making more likely the contact of the inorganic components with the water phase. Thus, the encapsulation of the inorganic species occurred using KPS, whereas for identical polymer–inorganic combinations, the use of AIBN and AMBN leads to phase segregation and the formation of Janus-like structures. The third parameter controlling the development of hybrid morphologies is related to the polarity of the different species in the hybrid and its essential role played in the interfacial energy of the whole system. The polarities of the polymer and the inorganic components determine the respective interfacial tensions with the aqueous phase (*γ*_PW_ and *γ*_IW_). For a particular polymer–inorganic combination, the differences of polarity can be tuned via the surface functionalization of the inorganic components. Comonomers are also used for compatibilization of the different species and the control of their self-assembly. Besides tuning the hydrophobicity, the functionalization of the inorganic surface is a versatile tool to introduce reactive groups, which can copolymerize and act as linkers between polymer and inorganic materials. The copolymerization allows for the control of the structure development, which cannot be simply explained with the model of González-Ortiz and Asua [51].

**Figure 3:**
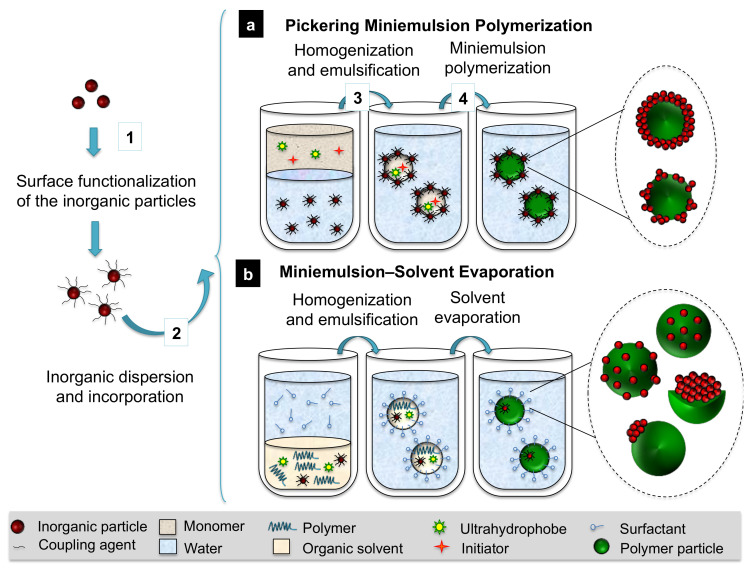
Scheme of the preparation of hybrid polymer–inorganic nanoparticles. Pickering miniemulsion polymerization (**a**) and miniemulsion–solvent evaporation (**b**) processes.

Besides the formulation of the miniemulsion, the synthetic strategy itself will also strongly determine the hybrid structure. As alternatives to the previously discussed cases of miniemulsion polymerization, Figure 3 introduces two further possibilities: Pickering miniemulsion polymerization and emulsion–solvent evaporation.

On the one hand, so-called Pickering or nanoparticle-stabilized emulsions, described for the first time by Ramsden in 1904 [55] and systematically studied by Pickering [56], are emulsions in which the molecular surfactants are substituted by a layer of nanoparticles irreversibly adsorbed at the droplet interface. Therefore, colloidal stability relies on the ability of the inorganic emulsifier to reduce the interfacial area between the liquid phases of the miniemulsion. This surfactant-free method has been used for the synthesis of hybrid nanoparticles by miniemulsion polymerization [57,58,59].

On the other hand, emulsion–solvent evaporation techniques are based on the use of preformed polymers, which are dispersed in a solvent for the preparation of the miniemulsion. The polymer precipitation occurs by further evaporation (e.g., by thermal methods or in supercritical conditions) and leads to the formation of polymer-based nanoparticles, among which the inorganic species can self-assemble. As explained in detail in the following sections, each strategy implies significant morphological differences, even for identical polymer–inorganic combinations. We seek to give insight into the morphology control reached via the synergic contribution of the synthetic strategy, the polymer–inorganic combination, and the miniemulsion formulation.

### 2.2. Preparation Strategies

#### 2.2.1. Hybrid Nanoparticles by Assembly of Preformed Polymer and Inorganic Components (Strategies Type A)

The deposition of inorganic species on the surface of preformed polymer nanoparticles has often been reported for clay platelets, which offer an almost complete coverage of different polymer materials, such as polystyrene, polyethylbenzene, poly(butyl acrylate) (PBA), or poly(methyl methacrylate) (PMMA) nanoparticles [60]. This strategy relies on miniemulsion polymerization processes using cationic surfactants, followed by the “layer-by-layer” complexation of silicates at the surface level. Furthermore, the external addition of tetraethoxysilane leads to its condensation with itself and with the remaining OH− groups from the clay sheets, resulting in the formation of an inorganic shell. Also by the assembly of preformed components, multiwall carbon nanotubes (MWCNTs) can be deposited on poly(styrene-*co*-butyl acrylate) particles [61].

The combination of solvent evaporation and miniemulsion techniques has allowed for the incorporation of iron oxides [62,63] and apatite [64] within poly(l-lactide) nanoparticles. For this aim, preformed polymer and inorganic nanoparticles dispersed in chloroform were used for the preparation of direct miniemulsions. The evaporation of the organic solvent by heating promotes polymer precipitation and formation of hybrid nanoparticles. The surface chemistry of the inorganic species plays an essential role in their rearrangement within the forming polymer matrix. The encapsulation of iron oxide nanoparticles and apatite was reached using oleic acid (OA) [62,63], and the amphiphilic block copolymer poly(butylene-*co*-ethylene)-*block*-poly(ethylene oxide) (PBE-*b*-PEO) as capping agent [64]. The ultrasonication time and the size and concentration of inorganic nanoparticles was shown to play an important role in the encapsulation performance, particle size, and inorganic load [63]. Moreover, multicompartment poly(vinyl formol)–titania nanoparticles with different morphologies were reached [65]. The successive phase separation processes between polymer and inorganic species with low affinity were controlled by the miniemulsion formulation. Pure Janus-like structures with two inorganic and polymer faces resulted from evaporation of chloroform. The addition of hexadecane resulted in an additional liquid compartment separated from the polymer by an interfacial layer of TiO_2_ nanoparticles. Supercritical CO_2_ has also been used as an alternative to thermal treatment for the extraction of chloroform used in the preparation of poly(lactic-*co*-glycolic acid)–magnetite nanoparticles [66]. In that case, the surface functionalization of magnetite with ricinoleic acid or poly(lactic acid) led to Janus-like structures or homogeneous distributions of magnetic clusters within the polymer matrix, respectively. The inorganic incorporation broadened the size distribution of the hybrid nanoparticles due to the competition between the emulsifier and the magnetite during sonication.

#### 2.2.2. Polymerization Processes in the Presence of Preformed Inorganic Components (Strategies Type B)

In the last decades, the preparation of superparamagnetic hybrid nanoparticles has been intensively studied for tracking, targeting, treatment, and diagnosis applications in biomedicine. In this context, magnetite nanoparticles have been encapsulated within different homopolymers and copolymers prepared by miniemulsion polymerization: poly(methyl methacrylate) (PMMA) [67,68,69,70], polystyrene (PS) [71,72], poly(styrene-*co*-methacrylic acid) (P(S–MA)) [71,73], poly(styrene-*co*-styrene sulfonate) (PS–SS) [74], poly(styrene-*co*-4-vinyl pyridine) (P(S–4-VP)) [74], poly(acrylic acid) (PAA) [75], poly(methyl methacrylate-*co*-ethylene glycol dimethacrylate) P(MMA–EGDMA) [76], and poly(methyl methacrylate-*co*-styrene-*co*-methacrylic acid) (P(MMA–S–MA)) [77]. In this type of strategies, the incorporation of inorganic nanoparticles required the hydrophobization of their surface to ensure compatibility with the monomer and forming polymer. Different degrees of encapsulation of magnetite were reached using oleic acid (OA) [67,68,69,76], ammonium oleate in acid media [71,72,73,74], or silane compounds such as 3-(methacryloyloxy)propyl trimethoxysilane (MPS) [70,75,77,78] and octadecyl trimethoxysilane (ODTMS) [78,79] as coupling agents.

**Figure 4:**
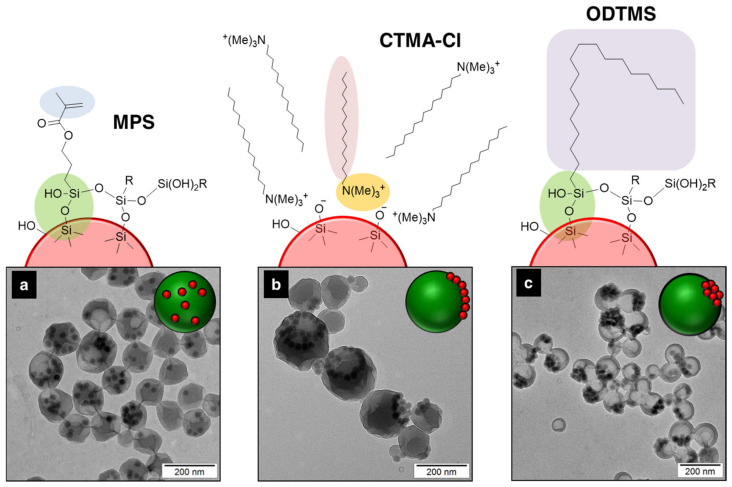
SEM images of poly(methyl methacrylate)–silica hybrid nanoparticles by miniemulsion polymerization. Role of the chemical structure of the functionalizing agent over the hybrid morphology: (**a**) 3-methacryloxypropyltrimethoxysilane (MPS); (**b**) cetyltrimethylammonium chloride (CTMA-Cl); (**c**) octadecyltrimethoxysilane (ODTMS). Created by the authors with own data from ref. [79].

Surface functionalization of preformed inorganic nanoparticles (e.g., silica [80,81,82,83,84,85] or magnetite [78]) via silane chemistry stands out as a versatile strategy to address the inorganic migration within a forming polymer matrix. The wide range of chain lengths and functional groups available among trialkoxysilane components offers more flexibility and effectiveness than the traditional carboxylic acids (e.g., OA) or ionic surfactants (e.g., CTAB). Figure 4 compares the efficiency of the condensation of the alkoxysilane components on the surface of silica nanoparticles with the ionic interaction between the negatively charged silica surface and the positively charged CTAB salt. The use of silanes with polymerizable vinyl moieties, such as MPS, allowed for the homogeneous encapsulation and fixation of metal oxide nanoparticles via copolymerization with the surrounding monomer (e.g., MMA [82,84], styrene and MMA [85], BA and MMA [83]). The use of silane components, such as ODTMS, with longer alkyl chains and no polymerizable units, generates thermodynamically preferred structures with the inorganic component at the surface of polymer particles or Janus-like morphologies [78,79,80,81].

In the case of polymer matrices based on mixtures of styrene and 4-vinyl pyridine (4-VP), the MPS-functionalized silica nanoparticles formed an inorganic core chemically bonded to polystyrene, whereas raw silica nanoparticles added post-sonication remained anchored to the polymer surface through acid–base interactions between the silanol group of silica and the amino group of 4-VP [86]. At high pH values, double-decked raspberry-like morphologies were formed. The combination of traditional coupling agents (e.g., OA) and silane components (e.g., MPS) was also reported as a synergic hydrophobization strategy that led to mono- or multicore structures depending on the sonication time [87]. Moreover, polymer-grafting strategies have also been used for inorganic (e.g., silica) surface functionalization. Alkoxyamine initiators with a functional group were used as an alternative to functionalizing agents, which allowed for the controlled growth of polystyrene chains on the silica surface. After miniemulsion polymerization, the polystyrene-grafted silica nanoparticles were entrapped within a polystyrene matrix. In this case, hybrid core–shell structures were achieved by adjusting the molecular weight of the grafted polymer [88].

Other authors have addressed the structural control of hybrid nanoparticles by tuning the size of the inorganic nanoparticles (e.g., silica) [79,89,90,91], the nature and the concentration of the surfactant [91], or by adding auxiliary comonomers [89,90,91,92]. The encapsulation of silica forming multicore structures was improved with the reduction of size of the inorganic nanoparticles, whereas raspberry-like or core–shell structures were achieved by modification of the surfactant concentration [90,91]. MPS-functionalized silica nanoparticles were incorporated within polystyrene [87,88,90] and PMMA [89] matrices, including auxiliary comonomers, such as 4-VP [86] or butyl acrylate (BA) [89,91]. The presence of soft comonomers with carboxylic groups (e.g., BA) favors the inorganic incorporation by reducing the surface energy of the nanodroplets due to structural similarity with the organomodified silica [92].

From an inorganic perspective, a wide range of species has been incorporated within polymer matrices prepared by miniemulsion polymerization processes. Examples of such inorganic components are TiO_2_ [93,94,95], CeO_2_ [96], ZnO [97], TiO_2_ and ZnO [98], Fe_2_O_3_ [99], Ag [100], Au [101], halloysite nanotubes (HNTs) [102], and graphene oxide (GO) [103,104,105]. Clay platelets were used in the preparation of poly(methyl methacrylate-*co*-butyl acrylate) (P(MMA–BA) hybrid nanoparticles for the development of films. According to the application, butyl acrylate, allyl methacrylate, stearyl acrylate, and *n*-dodecyl mercaptan were used as soft comonomer, cross-linking agent, costabilizer, and chain-transfer agent to control the cross-linking density and the adhesion properties. The larger dimensions of the clay platelets with respect to the miniemulsion droplets led to dumbbell-like structures with the clay platelets embedded between two polymer particles or located at the particle/water interface. The resulting hybrid morphology was determined by the functionalization of the clay platelets with (2-methacryloxyethyl)hexadecyldimethylammonium bromide or methyl-bis-2-hydroxyethyl tallow ammonium [106]. P(MMA–BMA) nanoparticles with clay platelets engulfed within the matrix were also prepared by a semi-batch miniemulsion polymerization process using a redox system (*tert*-butyl hydroxyperoxide and ascorbic acid) as initiators. A fresh inlet of monomers, stearic acid, boric acid (for auxiliary stabilization), and an oil-soluble initiator (e.g., AIBN) led to a seed-emulsion polymerization governed by droplet and micellar nucleation mechanisms. The strategy allowed for the increase in the solid content of the final dispersion to meet industrial requirements [107]. Nickel nanoparticles hydrophobized with oleylamine and triphenylphosphine were also incorporated within polystyrene and PMMA matrices. The hybrid morphology was related to the anionic (e.g., sodium dodecyl sulfate (SDS)) or non-ionic (e.g., poly(ethylene oxide) hexadecyl ether (Lutensol AT50)) nature of the surfactant, as well as the high (e.g., KPS) or low polarity (e.g., AMBN) of the initiator. Thus, the study revealed that the compatibilization with the monomer does not ensure the encapsulation of the inorganic nanoparticles within the polymer.

**Figure 5:**
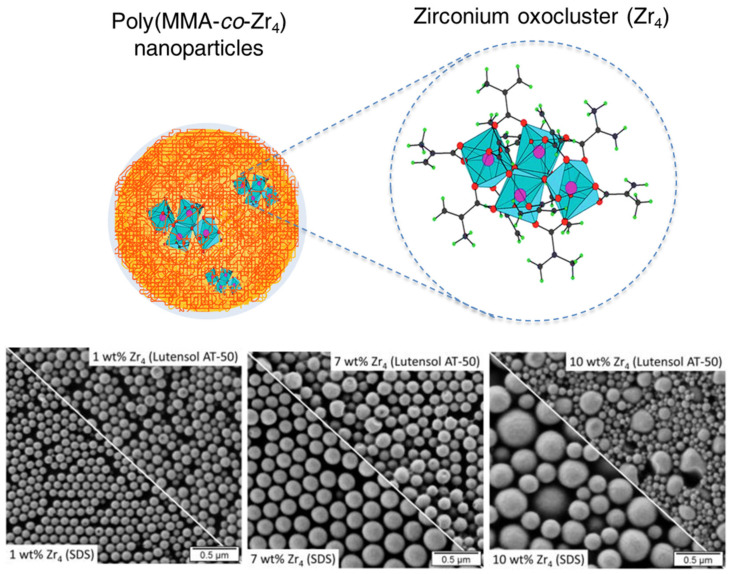
SEM micrographs of poly(MMA-*co*-Zr_4_) nanoparticles prepared using different concentrations of the zirconium oxocluster Zr_4_O_2_[O(O)CC(CH_3_)=CH_2_]_12_ (abbreviated as Zr_4_) using Lutensol AT50 as a surfactant. Figure created by the authors with own data from ref. [110].

By increasing the inorganic hierarchy level, β–diketonate metal complexes Me(tmhd) (tmhd; 2,2,6,6-tetramethyl-3,5-heptanedione) of Gd, Eu, Tb, La, Yb, Co, Cr, Al, Mn, In, Bi, Ga, Cu, and Ag were integrated within a PMMA matrix using different surfactants combinations [108]. The metal components were restricted via complex self-assembly processes in a bilayer conformation created between lamellar surfactants and the polymer matrix. The hybrid morphology was controlled with the chain length of the anionic surfactants, which determines the interlayer distance. Nano-onions or kebab-like structures were respectively formed when sodium alkylsulfates or dodecylphosphates, and sodium carboxylates were used as surfactants. Miniemulsion polymerization was also suitable for the incorporation of magnetic (Mn_12_O_12_(VBA)_16_(H_2_O)_4_ and Mn_8_Fe_4_O_12_(VBA)_16_(H_2_O)_4_) [109] and zirconium (Zr_4_O_2_[O(O)CC(CH_3_)=CH_2_]_12_, abbreviated as Zr_4_) [110,111] oxoclusters within polystyrene [109] and PMMA [110,111] matrices. The incorporation of the vinylbenzoate (VBA) ligand and vinyl groups in the hybrid structure of the oxoclusters enabled the copolymerization with the monomers. The hybrid morphology, cross-linking, and swelling degree of the final structure were related to the anionic (e.g., SDS) or non-ionic (e.g., Lutensol AT50) nature of the surfactant, the cluster content, and the reticulation of the polymer chains [110]. In this sense, Figure 5 demonstrates the role of the surfactant and the oxocluster concentration in the polydispersity of the system.

In contrast to direct miniemulsions, only a few examples have been reported on free-radical polymerization in inverse systems for the preparation of hybrid particles. Poly(2-hydroxyethyl methacrylate) (PHEMA) nanoparticles containing hydrophilic metal salts (combinations of metal cations (Fe^2+^, Fe^3+^, Co^2+^, Ni^2+^, Cu^2+^, and Zn^2+^) and BF_4_^−^, NO_3_^−^, and Cl^−^ ions) were prepared by miniemulsion polymerization in cyclohexane with AIBN as initiator [112]. The distribution of the salt within the polymer was mainly determined by electrostatic cation–ion interactions of the salt.

Pickering miniemulsions offer a surfactant-free platform for the preparation of hybrid nanoparticles via heterophase polymerization processes [113]. Nowadays, the technique is widely used in direct systems. Titania (TiO_2_) nanoparticles functionalized with acetylacetone and parabenzene sulfonic acid [114,115], and ceria (CeO_2_) nanoparticles were used as emulsifiers in the free-radical copolymerization of MMA or BA and MMA [116]. Using titania nanoparticles, homogeneously covered honeycomb nanostructures were achieved [115], whereas the irregular shape of ceria clusters hindered a complete inorganic coverage [116]. In Pickering miniemulsions, the wettability of the stabilizing inorganic nanoparticles at the oil–water interface is greatly affected by their concentration and surface chemistry, the pH value, or the ionic strength of the medium [113,117]. Therefore, the colloidal stability can be controlled by adjusting the amphiphilicity of the inorganic stabilizer through different functionalization strategies. In this fashion, poly(vinyl acetate-*co*-vinyl neodecanoate) latexes were prepared using silica nanoparticles modified with 2-[methoxy (polyethyleneoxy)propyl] trimethoxy silane as inorganic stabilizer [118,119]. The authors used the polyethylene oxide chains as a coupling agent, which allowed for Pickering stabilization but did not guarantee the stability of the final dispersion. At higher loadings of emulsifier, the particle size and the molecular weight of the polymer decreased. The initiator (i.e., hydrophobic or hydrophilic, ionic or non-ionic, and with thermal or redox activation) played a critical role on the final stability. While oil-soluble initiators, such as AIBN or lauroyl peroxide, provoked limited coalescence and partial secondary nucleation during the first stage of polymerization, water-soluble thermal initiators (either cationic, such as 2,2′-azobis(*N,N*′-dimethyleneisobutyramidine) dihydrochloride (ADIBA); anionic, such as potassium persulfate (KPS) or ammonium persulfate (APS); or uncharged redox initiators, such as tertbutyl hydroperoxide (TBHP) or Bruggolite 7 (BFF7)) led to unstable latexes due to droplet nucleation processes. Blank polymer particles (i.e., without inorganic species) resulted from the homogeneous nucleation triggered by the free-radicals in water [119].

During the early stages of the polymerization in nanoparticle-stabilized miniemulsions, the changes between the droplet/water and the forming particle/water interfaces can affect the metal oxide–polymer affinity, modify the adsorption energy of the inorganic component, and create slight coalescence problems. Therefore, the total conversion of the monomers was restricted and some bucket morphologies appeared due to the accumulation and further evaporation of the unreacted monomer [114,115,116]. In addition, some empty polymer particles (i.e., without inorganic species) were formed due to homogeneous nucleation processes generated by radicals from the aqueous phase [119]. Similar drawbacks were observed when clay disks were used as stabilizers in the polymerization of MMA or MA using water-soluble initiators [120]. Better results were achieved using hydrophobic monomers (e.g., styrene, lauryl methacrylate, butyl methacrylate, octyl acrylate or 2-ethyl hexyl acrylate), and dimethyl-2, 2-azobis(isobutyrate) as oil-soluble and non-ionic initiator [121]. The addition of sodium chloride was used to promote a slight colloidal instability, which compresses the double layer, creates partial flocculation, and increases the partitioning of the inorganic particles to the oil-water interface, where they reversibly adhere during emulsification. Nevertheless, some compartmentalization and intermediate monomer conversions were still found within the smaller nanoparticles.

The polymerization of styrene, benzyl methacrylate, and *t*-butyl methacrylate by miniemulsion polymerization using graphene oxide (GO) as a surfactant has also been reported [57,122]. Similarly, carboxylated graphene quantum dots (cGQDs) have been used in the miniemulsion polymerization of styrene to form fluorescent/photoluminescent nanocomposites [59]. In a related strategy, Zhou et al. [123] used γ-Fe_2_O_3_ particles functionalized with lignosulfonate as the only stabilizer in a Pickering miniemulsion polymerization of polystyrene without using any auxiliary comonomer or surfactant.

Although less common, Pickering inverse miniemulsions have also been employed for the preparation of hybrid nanoparticles. Interfacial polymerization processes are particularly common among colloidal synthesis in inverse miniemulsions. In a typical procedure, a hydrophilic monomer such as polyol is included in the disperse phase, while a more hydrophobic monomer is externally added. The partial solubility of the second monomer in the continuous phase promotes its diffusion towards the interface. The direct contact between monomers at the droplet interface leads to polymerization processes leading to the progressive formation of a polymer matrix from the outer to the inner side of the nanodroplet. Following this procedure, magnetite nanoparticles functionalized with OA were encapsulated within a poly(urea/urethane) matrix using 1,6-hexanediol and isophorone diisocyanate as monomers. The use of cordamol GTCC as costabilizer improved the inorganic incorporation at the expense of the increase in the particle size [124]. The surface of the hybrid resulting hybrid structure was further used for the immobilization of enzymes [125,126] looking forward to the development of enzymatic catalysts.

#### 2.2.3. Use of Preformed Polymers as Supports for In-Situ Inorganic Precipitation/Crystallization (Strategies Type C)

**Figure 6:**
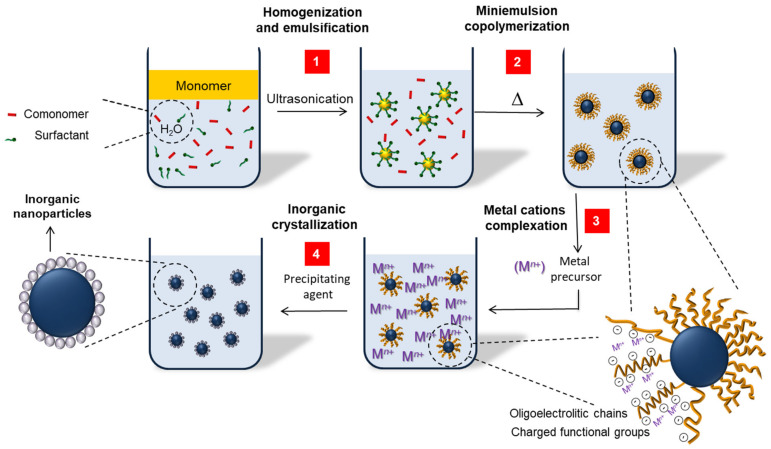
Schematic representation of the preparation of polymer/metal oxide hybrid nanoparticles by miniemulsion copolymerization and in-situ inorganic crystallization on the particle surface. Adapted with permission from [127] Mari, M.; Müller, B.; Landfester, K.; Muñoz-Espí, R. *ACS Appl. Mater. Interfaces* **2015**, *7*, 10727–1073. Copyright 2015 American Chemical Society.

The controlled crystallization of different metal oxides (e.g., ceria [127], maghemite (Fe_2_O_3_), magnetite (Fe_3_O_4_), zinc oxide (ZnO) [128], and CdS [129,130]) has been performed on polystyrene, PMMA, and polyacrylamide nanoparticles used as polymer supports for the preparation of hybrid nanoparticles with accessible inorganic functionalities. The polymer structures were produced by miniemulsion polymerization processes in aqueous or alcoholic (methanol, ethanol, and 2-propanol) media [128]. This type of synthesis requires the introduction of functional groups (e.g., carboxylic, phosphate, phosphonate or sulfate groups) accessible on the polymer surface to allow for the complexation of metal ions (e.g., Ce(IV) ions), as precursors of the final crystalline particles. Surface functionalization of polymers has been traditionally achieved by using available comonomers (e.g., acrylic acid [58,127,131], maleic acid [127], ethylene glycol methacrylate, phosphate vinylphosphonic acid [132], or vinylbenzylphosphonic acid) with different combinations of functional monomers and conventional surfactants [127]. After polymerization, the metal ions are incorporated in the system by external addition of a metal-based salt (e.g., cerium nitrate hexahydrate). The metal ions complexate on the polymer surface and, subsequently, metal oxide (e.g., ceria) nanoparticles are crystallized by controlled addition of a precipitating agent (e.g., sodium hydroxide). As a consequence, a layer of crystalline inorganic species assembles on the polymer surface. The resulting inorganic particles enter in competition with the molecular surfactant and may lead to a Pickering stabilization of the final dispersion. Figure 6 presents schematically the preparation of polystyrene-supported ceria nanoparticles using carboxylic and phosphate functional groups. In a similar way, the bioinspired mineralization of calcium phosphate was performed on differently surface-functionalized polystyrene nanoparticles. The mineralization process was achieved through a sequential addition of calcium (e.g., with Ca(NO_3_)_2_·4H_2_O) and phosphate (e.g., with (NH_4_)_2_HPO_4_) ions, sufficiently spaced in time to ensure the binding of the Ca^2+^ ions to the polymer surface [132]. In this case, the inorganic coverage and the crystalline morphology depended only on the negative charges present on the polymer surface, independently of the comonomer used. Thus, the concentration and the nature of the surfactant, together with the effect of the pH value and the functional comonomer, allowed for tuning the crystallinity, the morphology (needle-like or platelet) of the (if formed) hydroxyapatite crystals, and the density of inorganic material on the polymer surface [131].

**Figure 7:**
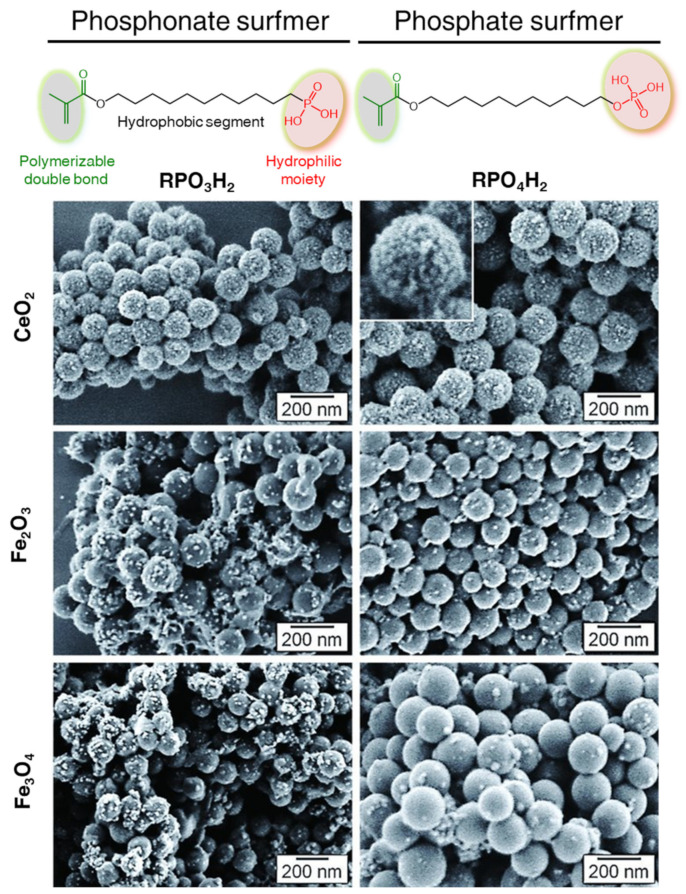
SEM images of phosphate- and phosphonate-functionalized polystyrene nanoparticles covered by metal oxides (CeO_2_, Fe_2_O_3_ and Fe_3_O_4_) species crystallized in aqueous media. The structure of the used functional surfmer is shown above. Based on ref. [128].

Alternatively, incorporation of functionalities (e.g., phosphate or phosphonate groups) on the polymer surface by combinations of comonomers and traditional surfactants has been achieved using polymerizable surfactants, so-called surfmers [128]. In this approach, the complexation chemistry plays a critical role in morphology control. A dense and homogeneous coverage of the polystyrene nanoparticles functionalized with phosphate and phosphonate surfmers was achieved with the crystallization of CeO_2_ nanoparticles in aqueous media. In contrat, as shown in Figure 7, the use of iron oxide (maghemite or magnetite) nanoparticles yielded raspberry-like structures. The use of alcohol in the continuous phase allowed for the development of raspberry-like morphologies with ZnO and improved the surface density of the inorganic material with Fe_2_O_3_ via a sol–gel-like oxide formation. Bulk inorganic crystallization occurred when using sulfate-based surfmers and a hydrophilic initiator (i.e., KPS).

The biomimetic precipitation of calcium phosphate has also been reported using biopolymer templates (e.g., poly(l-lactic), poly(ε-caprolactone), and poly(d,l-lactide-*co*-glycolide)), whose surface charge results from a combination of miniemulsion, solvent evaporation, and hydrolysis processes [64]. The preformed polymer dissolved in chloroform was subjected to a certain degree of degradation via random saponification during the ultrasonication (i.e., emulsification). Following solvent evaporation, sodium hydroxide was added for hydrolysis purposes. The resulting anionic functional groups (i.e., carboxylic and hydroxyl groups) on the polymer surface allowed for the mineralization of calcium phosphate structures. Among the inorganic species formed, the coexistence of amorphous forms of calcium phosphate and crystalline hydroxyapatite phases was observed.

#### 2.2.4. “All-In-Situ” Formation of Hybrid Nanoparticles (Strategies Type D)

What we name as “all-in-situ” strategies are associated with hydrolysis and condensation reactions that lead to the formation of inorganic species simultaneously with miniemulsion polymerization. These processes can occur in direct (O/W) [133,134]) or in inverse (W/O) [135,136,137]) miniemulsion systems. Following this strategy, the synthesis of silica nanoparticles during polymerization processes has been reported using tetraethyl orthosilicate (TEOS) as precursor whose hydrolysis and condensation under basic conditions was promoted with the external addition of ammonia [138] or triethylamine [139]. The base was added half an hour after the beginning of the polymerization to allow for a morphological pre-arrangement, depending on the type of initiator and the differences in affinity between the silica precursor and the monomers/polymers. For instance, the initiation of the copolymerization of methyl methacrylate (MMA) and butyl acrylate (BA) by AIBN caused compression of nodules of the silica precursor out of the nanodroplet confinement leading to raspberry-like morphologies. Furthermore, the in-situ functionalization of the forming inorganic particles with silane components (e.g., MPS) resulted in specific morphological features depending on the polymer matrix: P(MMA/BA), polystyrene [138], or poly(styrene-*co*-divinylbenzene) P(S/DVB) [139]. The use of the copolymerizable silane coupling agent led to an inorganic shell chemically bonded to the P(MMA/BA) surface [138]. A similar morphology was achieved in the copolymerization of styrene and DVB initiated by a redox complex (e.g., cumene hydroperoxide (CHPO) and tetraethylenepentamine (TEPA)), which diffuses from the continuous phase until the droplet interface, and in the presence of polystyrene as costabilizer [139]. The polymerization of styrene initiated mainly from the droplet interface using a water-soluble initiator (e.g., KPS) generated Janus-like structures. The increase in viscosity at the forming polymer shell controls the phase separation and relocation of the silica precursor and the later formed nanoparticles at one side of the structure. In this case, the silane agent was responsible for the chemical interaction between both hemispheres [138].

Polystyrene–titanium dioxide hybrid latexes with low encapsulation and a high inorganic loads (60–80 wt.%) were prepared using a similar strategy [140]. The titania precursor: tetra-n-butyl titanate (TBT) was included within the disperse phase of a direct miniemulsion with styrene and a hydrophobic initiator (AIBN). According to its hydrophilic and anionic nature, the inorganic precursor diffuses towards the oil–water interface where it undergoes an electrostatic interaction with a cationic emulsifier leading to TiOH, and further TiO_2_ nanoparticles via a sol–gel process. The presence of a chelating agent (e.g., acetylacetone) retarded the formation of the metal oxide and allowed for a certain inorganic encapsulation within the polymeric matrix. In addition, the use of DVB as a cross-linker improved the spherical morphology of the hybrid nanoparticles at the expense of reducing the inorganic coverage.

#### 2.2.5. Multistep Preparation of Hybrid Nanoparticles

The synthetic alternatives allowed in miniemulsion conformation are expanded by the combination of different strategies into complex multistep processes. To include those cases in this review, we have defined strategy types (X–Y), intermediates between two of the four main groups, or resulting from a sequential.

- *Assembly of Preformed Polymer–Inorganic Components Combined with Polymerization Processes (Strategies Type A–B)*

**Figure 8:**
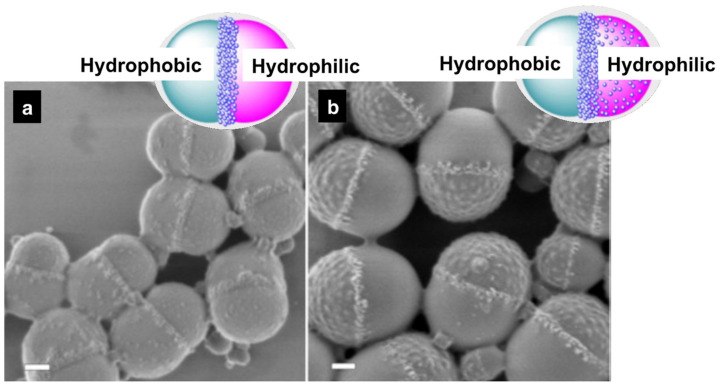
SEM micrographs and scheme of the multicompartment (**a**) and Janus-like (**b**) structures of poly(vinyl formal)–poly(dodecylmethacrylate)–titania nanoparticles (scale bars = 200 nm). Reprinted from [65] *Polymer*, vol. 55, Lv, L.-P.; Zhao, Y.; Zhou, H.-X.; Landfester, K.; Crespy, D., “From core–shell and Janus structures to tricompartment submicron particles” 715–720, Copyright (2014), with permission from Elsevier.

The preparation of multicompartment poly(vinyl formal)/TiO_2_ nanoparticles by solvent evaporation was combined with the miniemulsion polymerization of a second monomer (e.g., dodecylmethacrylate (DMA)) using divinylbenzene (DVB) as cross-linking agent [65]. The evaporation of chloroform allowed for a pre-arrangement into a tricompartment structure, with the monomer restricted to one side. Further polymerization of DMA led to solid tricompartment structures or Janus particles with two polymer faces (one elastomer and one amorphous) separated by an inorganic interface. The surfactant and comonomer content allowed for controlling the distribution of the TiO_2_ nanoparticles, as observed in the SEM micrographs in Figure 8.

An inverted sequence of the process was also reported for the preparation of PMMA–TiO_2_ nanoparticles surrounded by a second outer SiO_2_ shell. In this case, preformed MPS-functionalized TiO_2_ nanoparticles were encapsulated in a polymer matrix by miniemulsion polymerization of MMA. The polymer surface was further covered with preformed silica functionalized with γ-glycidoxypropyltrimethoxysilane through inorganic deposition. Pure core–shell structures were achieved by controlling surface chemistry of the inorganic components and the content of the hydrophobe (e.g., hexadecane) and the costabilizer (e.g., acrylic acid) [141].

- *Inorganic Synthesis and Polymerization Processes in the Presence of Preformed Counterpart Species or their Precursors (Strategies B–C)*

The co-homogenization of multiple inverse miniemulsions, consisting in aqueous solutions of inorganic precursors dispersed within a common polymerizable continuous phase, has been used to further prepare a direct miniemulsion where the polymerization takes place. Beyond the interest in the mechanistic process, the strategy is valuable to reduce the use of organic solvents. The technique has been reported for the incorporation of inorganic pigments with poor solubility in water (e.g., zinc phosphate [142], calcium carbonate, or barium sulfate [143]) into complex polymer–epoxy matrices with an adjustable composition of vinyl monomers. Similarly, silane-functionalized silica nanoparticles were incorporated within a P(MMA/S) matrix. The hydrolysis and condensation of the inorganic precursor (e.g., TEOS) under acid or basic conditions was driven by the co-homogenization of two inverse precursor miniemulsions. The hybrid morphology was controlled by the acid or basic nature of the catalyst, which determined the mechanism of formation of silica. Silica nanoparticles were entrapped within the polymer under acidic conditions, whereas a basic environment promoted the selective migration of the inorganic particles towards the interface. Other thermodynamic (e.g., interaction between the surfactant and the forming silica species) and kinetic (e.g., viscosity) factors also influenced the morphology development [144].

The sequential incorporation of different inorganic species was reported for the preparation of polystyrene-based multifunctional nanoparticles with a magnetic core and cadmium sulfide (CdS) nanoparticles embedded at the surface forming a raspberry-like structure. Two direct precursor miniemulsions were co-homogenized allowing for the incorporation of oleic acid-capped magnetite nanoparticles within the disperse phase with styrene as main monomer. The copolymerization of styrene and a surfmer (i.e., 11-(methacryloyloxy)undecylphosphonic acid (RPO_3_H_2_)) yielded phosphonate-functionalized latex nanoparticles. The functional polymer surface served as complexation template for the immobilization of Cd^2+^ ions introduced with the external addition of a precursor. Then, the crystallization of CdS nanoparticles was promoted by a controlled addition of a precipitating agent (e.g., sodium sulfide nonahydrate (Na_2_S·9H_2_O)) [129]. Similarly, magnetite and silica nanoparticles were sequentially incorporated within a polystyrene matrix. In this case, neither polymer functionalization nor use of surface-active monomers were required [145]. First, preformed oleate-capped iron oxide nanoparticles were encapsulated within polystyrene nanoparticles by heterophase polymerization in direct miniemulsion. The resulting Janus-like structures were used for the preferential hydrolysis and condensation of TEOS on the inorganic region. The selective formation of the silica coating caused the progressive formation of a cavity at the polymer side. Thus, smaller iron oxide/polystyrene nanoparticles could assemble by shape complementarity providing a certain colloidal steric stabilization. Such unusual anisotropic assembly remained fixed by the ongoing formation of a second siloxane network around them, which resulted in an irreversible bicomponent self-arrangement.

A combination of miniemulsion polymerization and sol-gel processes was also applied in the preparation core–shell poly(2-hydroxyethyl methacrylate) poly(HEMA)–silica nanoparticles containing iron or cobalt tetrafluoroborated salts. The process was mediated by the interaction between the metal salt and the silica precursor (TEOS). The polymer template was used for the interfacial deposition of the silica species. The control over the salt concentration allowed for controlling the transition between hybrid nanoparticles or capsule morphologies. The final conversion of the metal salts into metal oxides by calcination led purely inorganic hollow silica particles, which remain beyond the scope of this review [146].

In contrast to other commonly used strategies, inverse miniemulsion provides a versatile environment to perform multistep processes. Silver nanoparticles have been widely incorporated within biocompatible polymer nanoparticles by a combination of free-radical polymerization and reduction of a metal precursor salt. Silver tetrafluoroborate (AgBF_4_) salt was used as an osmotic agent in inverse miniemulsions stabilized with block-copolymers for the copolymerization of 2-hydroxyethyl methacrylate (HEMA) [144] methacryloyloxyethyl phosphorylcholine (MPC) [147]. The reduction of Ag^+^ ions to Ag^0^ was further achieved by diffusing gaseous hydrazine, creating certain swelling and increase in the particle size [147]. The slow diffusion rate of hydrazine and the fast reduction of Ag^+^ ions diffusing towards the polymer surface formed raspberry-like structures [144,147]. In this case, the molecular weight of the surfactant was proven to have an essential role over the colloidal stability and the size of the silver nanoparticles [144]. Inverting the sequence of the steps, the reduction of a metal precursor salt (i.e., silver nitrate (AgNO_3_)) via a polyol process followed by the free-radical polymerization reaction of 1-vinyl-2-pyrrolidone at high temperature, led to raspberry-like nanohybrids [148].

Inverse miniemulsions also allowed for the cross-linking of poly(vinyl alcohol) and glutaraldehyde under acidic conditions in the presence of Fe^3+^/Fe^2+^ precursor salts, followed by the external addition of a precipitating agent (i.e., triethylamine) [149]. A double-directional diffusion process of the metal ions and the precipitating agent towards the recently formed polymer–liquid interface ensured the coprecipitation of magnetite nanoparticles at the surface of the polymer nanospheres.

### 2.3. Applications of Various Nanoparticle Morphologies

**Figure 9:**
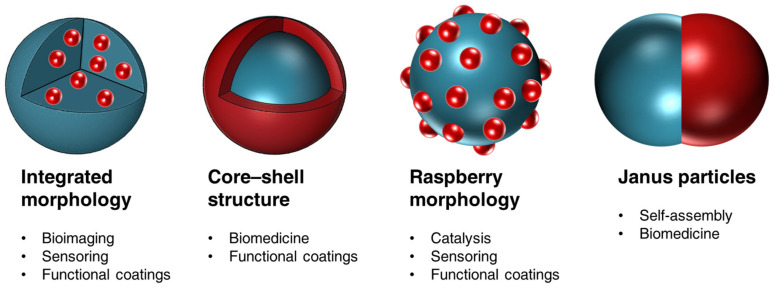
Scheme of some representative applications of different morphologies of polymer–inorganic hybrid nanoparticles, depending on the distribution of the inorganic and organic phases in their structure. The inorganic component is represented in red, while the polymer is represented in blue.

Polymer–inorganic nanomaterials formed by miniemulsion polymerization offer a wide range of applications in fields such as biomedicine [150,151], electronics [152], materials engineering [153], improvement of energy efficiency [154,155,156], and environmental protection [157]. The key factor that determines their field of application is not as much the method of formation, but rather the distribution of inorganic and organic phases within the nanomaterial. Specifically, what most influences their functionality is whether the inorganic part is encapsulated inside, which isolates and protects the material from the environment, or on the surface, allowing for greater interaction with the surroundings. Figure 9 illustrates a few representative applications of polymer–inorganic hybrid nanoparticles, depending on the distribution of the inorganic and organic phases in their structure. When the inorganic part is encapsulated, it remains protected, and the resulting hybrid is ideal for applications such as bioimaging [77]; luminescent [129], optical [158], and magnetoresponsive materials [159]; controlled release [160]; thermal energy storage [161]; and functional coatings (e.g., in construction [162] or against biodeterioration [163]). On the other hand, if the inorganic part is exposed on the surface, the nanomaterial interacts more with its environment, making it suitable for applications in catalysis [114,127,164], sensors [165], biocide materials [94,166,167], drug release [168], pheromone delivery [169], dental resins [170], photodegradation of organic contaminants [93,94,171,172], magnetoresponsivity [173,174], adhesives [175], anti-scratch transparent polyacrylic films, and different functional coatings [92,103,115,176].

## 3. Preparation of Hybrid Nanocapsules

### 3.1. General Overview

Miniemulsions can be used for the preparation of polymer–inorganic hybrid nanocapsules, a specific type of nanoparticles characterized by the presence of a solid shell occluding an inner core (e.g., a liquid). The capsule structures present a great potential for a wide range of applications (e.g., as hermetic encapsulating units, tracking carriers, or as vectors for the controlled release of substances). The combination of the polymer and inorganic features enhances the appeal of hybrid nanocapsules for the development of self-healing coating films [177,178], energy storage materials [179], or drug delivery systems applied in biomedicine [180].

The synthetic methods allowing for the achievement of a capsule-like morphology are divided into phase separation strategies and interfacial processes involving polymerization reactions (i.e., polymerization, polyaddition, and polycondensation) and/or inorganic synthesis (i.e., crystallization and hydrolysis/condensation of inorganic precursors) [44,181].

#### 3.1.1. Basic Principles for Structure Control of Hybrid Capsules via Phase Separation

The development of a capsule morphology by phase separation mechanisms is governed by similar physical principles to the ones described for hybrid nanoparticles. The minimization of the overall energy of a system consisting of three immiscible liquid phases (*i*, *j*, and *k*) is defined as the driving force for the achievement of the liquid core–shell structure [56]. The mathematical model proposed by Torza and Mason [182] is applied to explain the formation of capsules according to the spreading coefficient of each liquid phase (*s_i_*):

(2) $$s_{i}=\gamma_{\mathrm{jk}}-\left(\gamma_{\mathrm{ij}}+\gamma_{\mathrm{jk}}\right)$$

For a particular phase *i*, *s_i_* is determined with the contribution of the interfacial tensions (*γ_ij_*) between the different phases, as expressed in Equation (2). If in a direct miniemulsion the oil phase to be encapsulated is named as phase 1 (e.g., the hydrophobe), the continuous polar phase as 2 (e.g., an aqueous solution of a surfactant), and the oil phase precursor of the solid shell as 3 (e.g., monomer), different possible situations can be predicted by the model.

**Figure 10:**
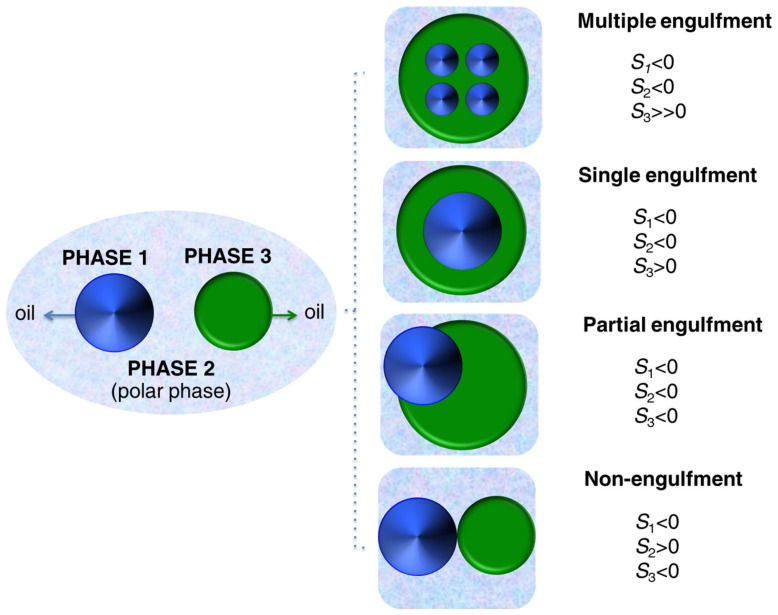
Phase segregation in a three-component system comprised of two oil dispersed phases (1 and 3) and a polar continuous phase (2), determined by the spreading coefficients (*s_i_*). Image drawn by the authors based on the model proposed by Torza and Mason [182].

- If the hydrophobicity of phase 3 is much higher than phase 1 (*γ*_12_ >> *γ*_23_) and the interfacial tension between the oil phases is low (Figure 10), a multiple engulfment of oil 1 in 3 is achieved.
- A core–shell morphology is reached by the single engulfment of the phase 1 into 3 when oil 3 is more hydrophobic than oil 1 (*γ_12_* > *γ_23_*) and the interfacial tension between the oil phases is still low.
- If phases 1 and 3 present similar hydrophobicity (γ_12_ ≈ γ_23_) and the interfacial tension between them is low, one of the oil phases (1) is partially engulfed by the other one (3) (Figure 10).
- If the interfacial tension between two oil phases (1 and 3) with similar polarity is high, non-engulfment and separation of the droplets as in Figure 10 occur.

Only the single engulfment of a second liquid phase within the nanodroplets of the miniemulsion (Figure 10) leads to the capsule morphology. Concerning the morphology development of the hybrid capsules, the process is again considered a result of the minimization of the overall energy of the system (*E*), expressed in terms of interfacial energies. Therefore, the energy balance of hybrid nanocapsules prepared in a direct miniemulsion is understood as the sum of the energetic contribution of four phases (i.e., the inorganic component (I), the aqueous phase (W), the monomer/dissolved polymer system (P), and the oil phase to be encapsulated (O)):

(3) $$E=\sum_{\mathrm{ij}}A_{\mathrm{ij}}\gamma_{ij}=A_{\mathrm{PW}}\gamma_{PW}+A_{IW}\;\gamma_{IW}+A_{IP}\gamma_{IP}+A_{OW\;}\gamma_{OW}+A_{\mathrm{OP}}{\;\gamma }_{\mathrm{OP}\;}+A_{\mathrm{OI}}{\;\gamma }_{\mathrm{OI}}$$

The spreading coefficient drives the phase separation of the liquid phases from the initial miniemulsion, among which the inorganic components assemble depending on the relative interfacial energies with the different liquids. After polymerization or solvent evaporation processes, the formation of a solid polymer shell leads to the most favorable hybrid structure with minimal interfacial energies [183].

**Figure 11:**
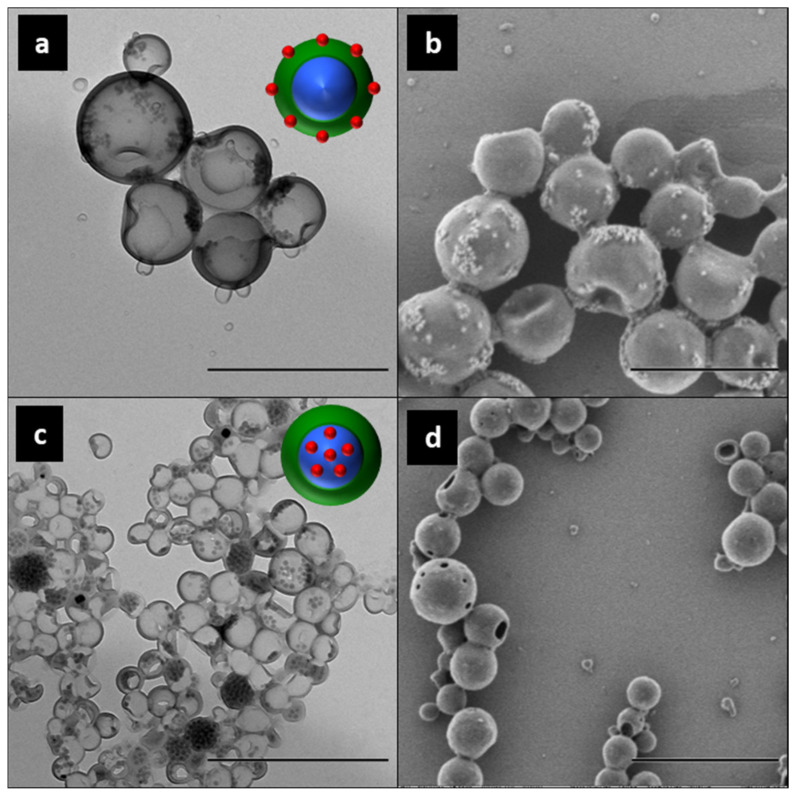
TEM (**a**,**c**) and SEM (**b**,**d**) micrographs of PMMA-based hybrid nanocapsule incorporating silica surface-functionalized with MPS (**a**,**b**) or ODTMS (**c**,**d**) (scale bar = 1 µm). Created with own research data related to ref. [79].

Polymerization processes have been commonly performed with an increased content of the hydrophobe phase for the preparation of capsules in direct miniemulsions via phase separation mechanisms. As an example, hydrophobized silica nanoparticles were encapsulated within PMMA nanocapsules with a hexadecane core using the solvent evaporation approach. Figure 11 presents the hybrid structures achieved depending on the surface functionalization of the silica nanoparticles [79,184]. The chemical similarity between the surface of the MPS-functionalized silica and the monomer/polymer system resulted in a low interfacial tension between phases and the assembly of the inorganic species within the PMMA shell. The higher affinity of ODTMS-functionalized silica nanoparticles for the hexadecane phase led to the preferred retention of the inorganic system within the liquid core of the capsules. Sundberg and Sundberg [185] carried out a complex theoretical and empiric study based on the morphological development of a system consisting of three organic phases inside an emulsion droplet. However, the complexity of the control of such systems is beyond the scope of this review. Double emulsions and solvent evaporation strategies have also been commonly used for the encapsulation of inorganic nanoparticles (e.g., magnetite) [186,187] within different polymer capsules.

#### 3.1.2. Capsule Formation via Chemical Processes at the Interface

The droplet interface of miniemulsions offers a platform for the preparation of hybrid polymer–inorganic nanocapsules. Both the organic and/or the inorganic components from the hybrid structure can be synthesized via interfacial processes. This type of strategy has been mostly exploited in inverse miniemulsions. From a polymer perspective, the synthesis of hybrid capsules has been commonly related to polyurea, polyurethane [188], or biopolymers (e.g., potato starch) [189] shells formed via interfacial polyaddition or polycondensation of diisocyanates using low-molecular-weight diamines or diols in the presence of inorganic nanoparticles. The inorganic species of hybrid nanocapsules can be synthesized via sol–gel processes taking place at the droplet interface of miniemulsions. In this fashion, the synthesis of silica nanocapsules has been commonly reported using TEOS, a traditional silica precursor, whose hydrolysis and condensation reactions are driven at the droplet interface under acidic or basic conditions [190,191]. Similar interfacial sol–gel processes have been reported for the preparation of nanocapsules with metals from group 4. In those cases, the metal precursor present in the nanodroplet was precipitated by the controlled addition of a base soluble in the continuous phase and partially soluble in the disperse phase (e.g., triethylamine) [192].

### 3.2. Preparation Strategies

#### 3.2.1. Hybrid Nanocapsules by Assembly of Preformed Polymer and Inorganic Components (Strategies Type A)

Thermo- and pH-responsive hybrid nanocapsules have been prepared via the heterocoagulation of commercial silica nanoparticles on the surface of poly(N-isopropyl acrylamide-*co*-*N,N′*-methylene bisacrylamide-*co*-4-vinyl pyridine) nanocapsules [193]. The polymer nanocapsules were synthesized by the copolymerization of isopropyl acrylamide (NIPAM) with *N,N*′-methylene bisacrylamide (MBA) and 4-vinyl pyridine (4-VP) in an aqueous miniemulsion. The monomers were responsible for the thermal sensitivity (NIPAM), the robustness (MBA) and pH-responsiveness (4-VP) of the system. The formulation of the polymer matrix allowed for the transition between nanocapsules and solid nanoparticles by substituting 4-VP with other functional monomers (e.g., acrylic acid (AA)).

Pickering stabilization of miniemulsions has also allowed for the preparation of synthetic capsule-like frameworks with a hybrid polymer–inorganic interface surrounding the aqueous confinement of the nanodroplets that could be used as nanoreactors or nanocarriers. In this fashion, hydrophobized silica nanoparticles were used to stabilize inverse miniemulsions with the aid of neutral copolymers (e.g., poly(ethylene-*co*-butylene)-*block*-poly(ethylene oxide), polyisobutylene succinimide pentaamine (sometimes referred in some literature as “Lubrizol U”), lecithin, or polyglycerol polyricinoleate (PGPR)) [194]. The versatility of the strategy was proven for different particle sizes, inorganic species, and different types of osmotic agents or emulsifiers.

#### 3.2.2. Preparation of Hybrid Nanocapsules via Polymerization Processes in the Presence of Preformed Inorganic Components (Strategies Type B)

Type B strategies are the most versatile methodology for the preparation of polymer–inorganic hybrid nanocapsules. Direct oil-in-water miniemulsions (O/W) have served for the synthesis of core–shell structures such as poly(S–BA)/SiO_2_ latex [195] or poly(HEMA–MDI)/SiO_2_ nanoparticles [196], poly(MMA–BA–AA–AM)/SiO_2_ nanocomposites [197], polypyrrole/indium tin oxide [198], poly(styrene-*co*-butyl acrylate-*co*-acrylic acid)/TiO_2_ systems [199], TiO_2_/polystyrene/Fe_3_O_4_ multifunctional nanocomposites [114], or PMMA/Fe_3_O_4_ nanoparticles [200]. Moreover, poly(NIPAM)/ZnO nanospheres were achieved by inverse Pickering miniemulsion polymerization [137]. In the same way, Cao et al. prepared raspberry-like nanocapsules of P(S–4-VP) with silica nanoparticles embedded within the polymer shell, with divinylbenzene used as a cross-linking agent [201]. The colloidal stability of the system relied on the interaction between a commercial silica sol and 4-VP moieties, which was highly influenced by the extension of the polymerization to the aqueous phase, the pH value, and the size of the silica particles. Hydrophobized silica or magnetite nanoparticles were encapsulated within PMMA nanocapsules with a hexadecane core using the solvent evaporation approach. The morphology control was achieved through the chemical structure of the silane used as coupling agent and its affinity to the polymer shell.

**Figure 12:**
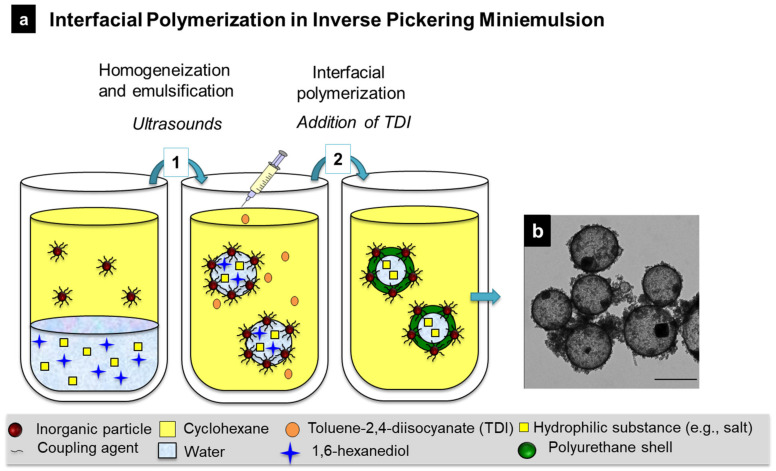
(**a**) Scheme of the preparation of polyurethane–silica submicron capsules by interfacial polymerization process in inverse Pickering miniemulsion; (**b**) SEM image of the hybrid capsules containing 2.5 wt.% of sodium chloride salt (scale bar = 1 µm).

Interfacial polymerization/polyaddition reactions are commonly reported in inverse miniemulsions. In this fashion, polyurea–magnetite nanocapsules were prepared via an interfacial polyaddition reaction between 1,6-diaminohexane (HMD) and toluene-2,4-diisocyanate (TDI). The amine moieties of polyisobutylene-succinimide pentaamine, used as a surfactant, allowed for the control of the partial reaction of TDI according to its ratio with the monomer. Magnetite nanoparticles were embedded within the polymer shell as a result of the phase segregation occurred during the polymerization [188]. A similar interfacial polyaddition reaction between 1,6-hexanediol and TDI in cyclohexane allowed for the preparation of polyurethane–silica hybrid capsules with an aqueous core. The surface of commercial silica nanoparticles was tuned with trimethoxy(propyl)silane (PTMS) to promote Pickering stabilization of the miniemulsion. Figure 12 schematically shows the encapsulation of inorganic salts through the formation of polyurethane–silica capsules by interfacial polymerization in an inverse Pickering miniemulsion. The hermeticity of the capsules allowed for the encapsulation of hydrated salts as phase change materials for thermal energy storage applications [202].

#### 3.2.3. Use of Preformed Polymers as Supports for the In-Situ Inorganic Precipitation/Crystallization (Strategies Type C)

**Figure 13:**
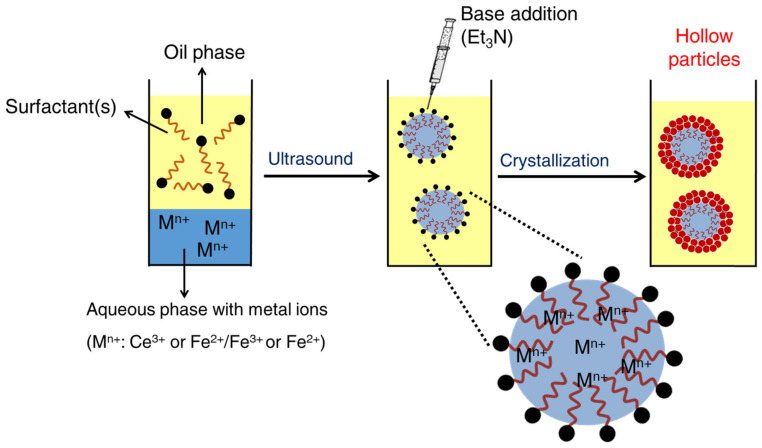
Schematic representation of the interfacial crystallization of metal oxides in inverse miniemulsion systems. Reprinted with permission from [199] Varol, H.S.; Álvarez-Bermúdez, O.; Dolcet, P.; Kuerbanjiang, B.; Gross, S.; Landfester, K.; Muñoz-Espí, R. *Langmuir* **2016**, *32*, 13116–13123. Copyright 2016 American Chemical Society.

Interfacial sol–gel processes have been performed in inverse miniemulsions for the preparation of inorganic nanocapsules, whose hybrid nature is given by polymeric surfactants used for colloidal stability. Nanocapsules of zirconia and/or hafnium oxides and hydroxides [88] were prepared by interfacial sol–gel precipitation processes using block copolymers (e.g., PGPR and Lubrizol) as surfactants. The strategy relies on the preparation of inverse miniemulsions including metal precursors (e.g., zirconyl chloride octahydrate (ZrOCl_2_·8H_2_O) or Hafnium(IV) oxychloride octahydrate (HfOCl_2_·8H_2_O)) within the aqueous phase. The prompt external addition of an organic base (e.g., triethylamine), soluble in the organic phase while having a limited solubility in the dispersed phase, led to the generation of OH^−^ groups at the droplet interface. The subsequent hydrolysis of the metal precursor forms metal hydroxo complexes. An inorganic polymeric network was generated by polycondensation reactions confined to the droplet interface. The inorganic species resulting from typical sol–gel conditions are amorphous and require calcination operations to become crystalline. Such aggressive thermal treatments would result into the degradation of the polymer, and thus, they are excluded in this review. The crystallization of other lanthanide oxides and transition metal oxides (e.g., CeO_2_, CuO, γ-Fe_2_O_3_, YCrO_3_) [203,204,205] can occur at relatively mild conditions, even at room temperature using analogous strategies. The interfacial crystallization of metal oxides in inverse miniemulsion is schematically represented in Figure 13.

Similarly, silica nanocapsules have been prepared by interfacial hydrolysis/condensation of TEOS using cetyltrimethylammonium bromide (CTAB) and poly(ethylene-*co*-butylene)-*b*-poly(ethylene oxide) as surfactants. The miniemulsion droplets served as soft templates to catalyze the reaction under either acidic [191] or basic [190] conditions. In the first stages, the hydrolyzed and condensed silica species dissolve within the aqueous droplets. As the reaction progresses, phase separation occurs and the capsule morphology develops. The amphiphilic structure of the surfactants acts as morphology-directing agents by confining the silica species preferentially at the droplet interface. The reaction time had an essential role in the thickness and cross-linking of the shell. At high concentrations of the inorganic precursor and/or low amount of surfactants the internal viscosity increases. Therefore, the insufficient interaction between the emulsifier and the silica species led to the transition from nanocapsules to solid nanoparticles [190,191].

#### 3.2.4. All-In-Situ Synthesis of Hybrid Nanocapsules (Strategies Type D)

Miniemulsion polymerization processes in direct miniemulsion have been driven (quasi)-simultaneously to the hydrolysis/condensation reactions of the silica precursor (e.g., TEOS [206] or PEOS [207]) for the incorporation of silica nanoparticles within different polymer-based (e.g., PMMA [208], P(S/DVB) [187] and P(MMA/DVB) [209]) nanocapsules. The presence of a coupling agent (e.g., MPS) favored the polymer–inorganic compatibility, which allowed for controlling the hybrid morphology. The transition from bowl-type nanoparticles to raspberry-like nanocapsules was observed with the increase in the silane concentration [208]. The synthesis of Cu_2_O/PMMA nanocapsules via RAFT miniemulsion polymerization has also been reported, using the amphiphilic quaternized poly(2-(dimethylamino)ethyl methacrylate)-*b*-poly(methyl methacrylate) macro-RAFT agent (QPDMAEMA-*b*-PMMA) as the stabilizing agent [210].

**Figure 14:**
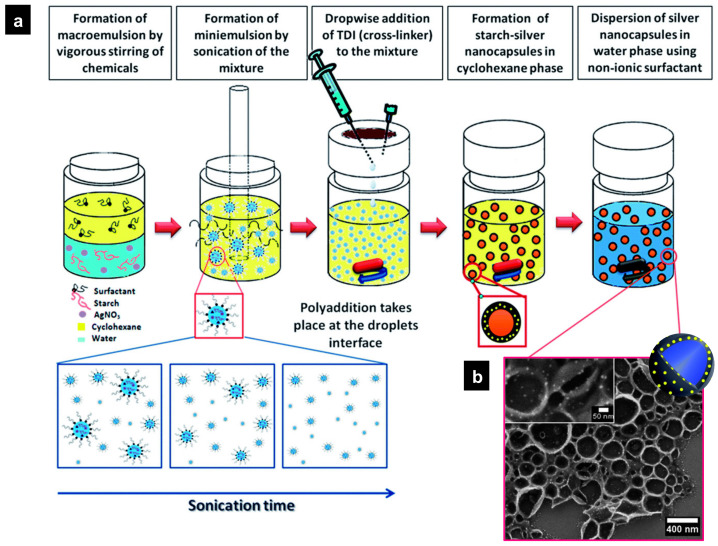
(**a**) Scheme of the preparation of starch–silver hybrid nanocapsules and (**b**) SEM micrograph of nanocapsules. Reproduced from ref. [189] (CC-BY license).

Interfacial polymerization and metal reduction processes in inverse miniemulsion were used in the production of biocompatible potato starch nanocapsules with embedded silver nanoparticles, as shown in Figure 14 [189]. The interfacial cross-linking reaction between the OH^–^ starch groups with the –NCO groups of TDI occurred during the reduction of a metal salt precursor (e.g., silver nitrate). The release of free radicals and d-glucose from the amylose of the starch led to the formation of metallic Ag nanoparticles. The shell thickness was controlled by the amount of isocyanate that was externally added. Similarly, the synthesis of CrCl/poly(methyl methacrylate) core–shell nanocapsules by inverse miniemulsion evaporation method has also been reported [211].

Poly(urethane/urea)–silica nanocapsules were prepared via an interfacial polyaddition process between the primary and secondary amines of a polyethyleneimine and the hydroxyl groups of a glycerol with TDI, simultaneous to the sol-gel precipitation of TEOS under basic conditions [181]. A cationic and hydrophilic surfactant was used to trap the negatively charged silicic acid Si(OH)_4_ to form silica capsules. The mixed regions of silica and poly(urea-urethane) without microphase separation gave rise to an interpenetrating structure with molecular pH-responsive gates. Additionally, the preparation of robust water/SiO_2_/polymer/SiO_2_ nanocapsules from a double miniemulsion system has also been reported, involving the conversion of the precursor into silica and the polymerization of the oil phase. Other intriguing nanostructures, such as Janus-like nanorattles and nanomushrooms, could be obtained by altering the preparation conditions. In particular, double emulsions of water-in-oil-in-water provide significant advantages over simple oil-in-water emulsions for microencapsulation, offering the ability to carry both aqueous and oily payloads and a sustained release profile [212].

#### 3.2.5. Multistep Synthesis of Hybrid Nanocapsules

- Inorganic Synthesis and Polymerization Processes in the Presence of Preformed Counterparts or their Precursors (Strategies Type B–C)

Shells of polystyrene and poly(acrylic acid) nanogels restricting a liquid core of hexadecane and coated with an outer layer of silica nanoparticles were prepared by a multistep inorganic–polymer synthesis in direct miniemulsion [213]. The hydrolysis/condensation of TEOS under the basic conditions created with the external addition of a base (e.g., triethylamine) to a direct pre-emulsion led to the synthesis of silica nanoparticles. The presence of MPS promoted the partial surface functionalization and preferential location of silica at the droplet interface. Applying high-shear forces resulted in a stable Pickering miniemulsion, where polymerization and phase separation processes occurred. The use of double (W/O/W) Pickering miniemulsions also allowed for the preparation of polystyrene nanocapsules with an aqueous core, incorporating amphiphilic silica nanoparticles at both the inner and outer water–polymer interfaces [214].

In this context, inverse miniemulsions were used for the preparation of double-shell polyurea–silver–poly(vinyl caprolactam) nanocapsules via a three-step synthesis [215]. Polyurea nanocapsules with an aqueous core containing silver nitrate salt were prepared by interfacial polyaddition. Then, the reduction of Ag^+^ ions to Ag^0^ nanoparticles was reached by gaseous hydrazine, followed by the creation of an outer polymeric amphiphilic shell by a free-radical polymerization process of 1-vinyl-2-pyrrolidone. The capsule size was controlled by the addition rate of the isocyanate and the surfactant content, whereas the thickness of the shell was determined by the monomer concentration. Poly(urethane/urea) capsules incorporating BaSO_4_ nanoparticles within the aqueous core were prepared similarly [216]. In this case, hardly soluble salts in water, such as sulfates, carbonates, phosphates, and oxalates of Ba^2+^, Ag^+^, Ca^2+^, or Pb^2+^ were formed through fusion and fission occurring during the co-homogenization of two miniemulsions containing water-soluble salts with the respective anions (e.g., Na_2_SO_4_) and cations (e.g., BaCl_2_). Afterward, the polymer shell was formed by the interfacial polyaddition of a polyol (e.g., glycerol or poly(vinyl alcohol)) and TDI.

**Figure 15:**
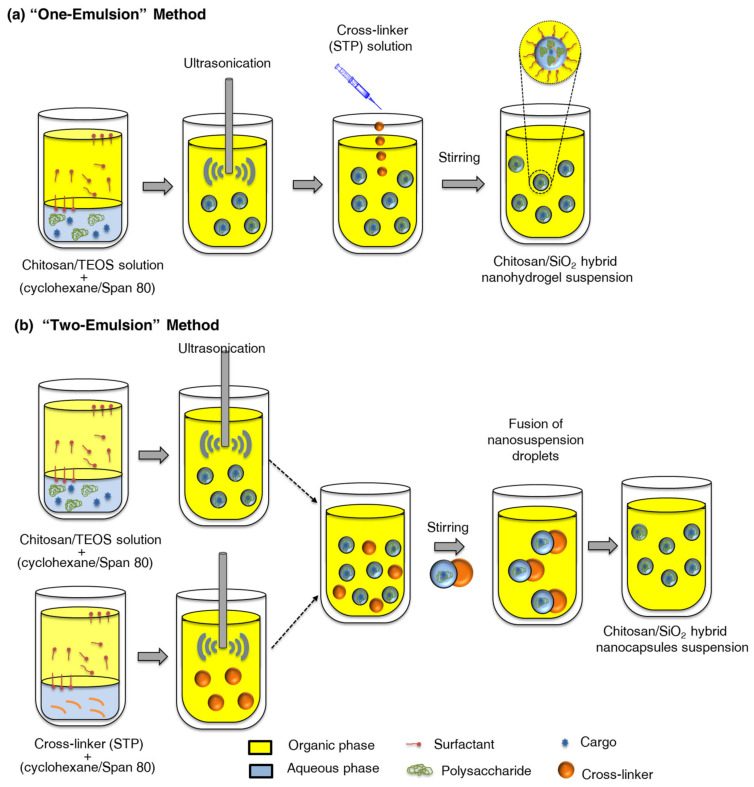
Schematic representation of the formation of cross-linked chitosan nanohydrogels encapsulating an active hydrophilic molecule through two methods: (**a**) a “one-emulsion” method and (**b**) a “two-emulsion” method (droplet-fusion method). Reproduced from ref. [217] (CC-BY license).

In a recent work by Elzayat et al. [217], chitosan/silica hydrogel nanocapsules were created through the ionic interaction between cationic groups in chitosan and anionic groups in sodium triphosphate (STP), which functions as a physical cross-linker. This work reported two different preparation methods, schematically depicted in Figure 15: the first involves adding STP to the continuous phase of an inverse emulsion of chitosan, while the second method combines droplets from two separate emulsions containing chitosan and STP. The resulting nanocapsules have sizes ranging from 50 to 200 nm. The efficiency of the hydrogel in encapsulating a hydrophilic model substance (erioglaucine disodium salt) was assessed for both methods by studying the release in a neutral aqueous medium. The results indicated that both approaches successfully encapsulate the hydrophilic substance, though the droplet-fusion method produces more stable suspensions. In general, the presence of silica in the systems consistently appeared to delay the release of erioglaucine.

- Simultaneous Inorganic and Polymer Synthesis, in the Presence of other Preformed Inorganic Species (Strategies Type B–D)

The simultaneous miniemulsion copolymerization and hydrolysis/condensation reactions of an inorganic precursor (e.g., TEOS for silica) were combined with the encapsulation of a second inorganic component preformed and functionalized nanoparticles (e.g., magnetite nanoparticles) [149,209]. The phase separation between the silica precursor (TEOS), the functionalizing agent (MPS), and the forming polymer restricted the silane compounds to a liquid core. The volume reduction that occurred with the formation of hydrophobic SiO_2_ nanoparticles covalently attached to the inner face of the polymer shell allowed for the capsule morphology [187,209]. In addition, “Janus-like” structures resulted from the application of an external magnetic field during the early phase of polymerization [187].

### 3.3. Applications of Nanocapsule Morphologies

Hybrid nanocapsules formed via miniemulsion offer a versatile solution for encapsulating, storing, protecting, and—when desired—releasing active substances in a controlled manner, whether hydrophilic or hydrophobic, thanks to a well-defined polymer–inorganic shell. These nanocapsules combine unique properties of different materials into a single particle, making them highly attractive for a wide range of applications. In the field of energy storage and management, for example, capsules with an organic–inorganic double shell enable efficient heat storage and release while responding to magnetic stimuli [218]. Similarly, bifunctional paraffin capsules coated with polyaniline/TiO_2_ not only store thermal energy but also facilitate CO_2_ photoreduction, making them a potential valuable tool in climate change mitigation [219]. Phase change materials encapsulated in organic–inorganic hybrid shells, such as those reported by Wang et al. [220], improve thermal stability and energy efficiency. Other studies have also shown that such capsules can also provide UV protection, making them ideal for coatings and surface protection [221].

In the coating technology, hybrid nanocapsules have been used for self-cleaning coatings [222] and for the controlled release of substances that promote self-healing. In the textile sector, hybrid nanocapsules have been applied to mitigate odors at high temperatures, enhancing fragrance retention in fabrics [223].

In the medical field, hybrid nanocapsules also play a crucial role. For instance, polyacrylamide/poly(methyl methacrylate)/silica hybrid microcapsules, obtained by inverse Pickering emulsions, have been proven to be effective in controlled drug release, taking advantage of their temperature-responsive properties [224]. Additionally, nanocapsules with magnetic particles in the shell, have been designed to target specific areas for controlled drug release [225]. Hybrid nanocapsules have also been employed in environmental protection, particularly in the absorption of heavy metals [226].

In summary, hybrid nanocapsules enable the development of innovative solutions across diverse sectors such as energy, biomedicine, industrial coatings, and textiles. They offer benefits including controlled release, surface protection, improved energy efficiency, and smart functionalities.

## 4. Conclusions and Outlook

Polymer–inorganic hybrid nanostructures, nanoparticles, and nanocapsules offer synergic combinations of the inorganic properties and polymer features, which are attractive for a wide range of sectors (e.g., catalysis and photocatalysis, pharmaceutic industry, energy storage, optoelectronics, or biomedicine). For decades, researchers have dedicated their efforts to the preparation of polymer–inorganic nanomaterials. Despite the significant advances that have been done in this area, the establishment of a pattern for morphology control of such complex nanostructures is still challenging. We offer an overview of the strategies that have been used for the preparation of polymer–inorganic hybrid nanoparticles and capsules in miniemulsion, and the variety of morphologies that has been achieved. We aim to provide a collection of cases of hybrid nanomaterials whose identical morphological development can be related to specific synthetic strategies in order to suggest general patterns of control.

Miniemulsion stands out as a synthetic platform that overcomes the structural limitations of other colloidal techniques. The theory governing the self-assembly of the different species of hybrid nanostructures (solid nanoparticles and nanocapsules) prepared in miniemulsion relates to the minimization of the interfacial energies of the system. The current models, however, are not able to provide the whole formulation of the miniemulsion, nor the processes required to reach a single structure. We suggest a combined approach to address morphology control via the selection of the synthetic strategy and the related operational parameters (e.g., polymer–inorganic species, surfactant concentration, or inorganic charge/load).

The preparation routes described in this review are classified into four main groups, depending on the spatial and chronological separation between the preparation of the inorganic components, the polymer and/or its precursors:

- *Group A* involves the self-assembly of preformed polymer and inorganic elements via inorganic complexation and solvent-evaporation techniques in miniemulsion. Complexation, heterocoagulation, or Pickering strategies result in the inorganic coverage of preformed polymer nanoparticles and nanocapsules. The formulation of the polymer matrix allows for the transition between capsule and particle morphologies. The solvent-evaporation method provides different degrees of encapsulation and phase segregation through specific inorganic surface functionalization.
- In *group B*, preformed polymers nanoparticles are used as supports for inorganic synthesis. The hybrid morphology is mostly controlled via the complexation chemistry between polymers and inorganic precursors, where the pH and the surfactant have an essential role. Inorganic precipitation and biomimetic mineralization result in “raspberry-like” structures, bulk crystallization, or homogeneous coverage of the polymer surface. Block-copolymers and non-ionic surfactants are also used as soft templates for the preparation of nanocapsules via interfacial sol–gel processes. The transition between capsules and particles is controlled by the inorganic precursor/surfactant ratio and the addition rate of the precipitating agent.
- *Group C* is based on polymerization processes in the presence of preformed inorganic components. The specific migration of the inorganic functionalities within a forming polymer matrix can be controlled by the nature/type of surfactant and initiator, and the differences of polarity of the monomer/polymer–inorganic system. The inorganic surface functionalization using coupling agents with a specific structure allows for a great variety of hybrid morphologies (e.g., homogeneous inorganic encapsulation, Janus, raspberry, or core–shell structures). Capsule morphologies are reported by an increased content of the hydrophobe in direct miniemulsions or interfacial polymerization and polyaddition processes in inverse miniemulsions.
- *Group D* comprises the most challenging and less explored strategies comprising quasi/simultaneous polymerization and inorganic synthesis processes taking place in miniemulsion. Miniemulsion polymerization or interfacial polymerization techniques have been combined with the hydrolysis/condensation of metal oxides or the reduction of metal nanoparticles. The morphology development of the nanohybrids follow the principles of reduction of the interfacial energy of the system. The attractiveness of this group relies on the minimization of the synthetic steps and the discovery of organic–inorganic interactions.
- Multistep processes have raised from the combination of individual strategies from the previous groups. The complexity and the potential of those syntheses are high both from the synthetic point of view and the achievable properties and morphologies of the final materials.

The novel synthesis of polymer–inorganic hybrid nanoparticles and nanocapsules is currently being directed towards the development of complex multifunctional nanostructures with a controlled accessibility of the functionalities. In addition, the progressive approach of nanotechnology to industry highlights the value of minimizing time and the number of process steps. The hierarchical attachment of nanoparticles of different natures is certainly a field to be further explored in the upcoming years, opening very interesting possibilities for the preparation of novel materials by assembly of functionalized nanoparticles. Moving beyond the state of the art in the synthesis of multifunctional nanoparticles will most likely be an important goal of the community in the near future. The systematic understanding of the key features determining the structure in multicomponent systems will also remain an active area of research.

## Figures and Tables

**Table 1:** Comparison of methods for preparation of polymer nanoparticles according to advantages and disadvantages, with indication of some relevant reviews for each case.

**Method**		**Advantages**	**Disadvantages**	**Refs.**
With polymerization	Conventional emulsion polymerization	- High-molecular-weight polymers - Good control over particle size - High reaction rates	- Presence of surfactants/stabilizers - Stability can be an issue	[2,3,4,5,6,7,8,9]
	Seeded emulsion polymerization	- Better control over particle size - Ability to core–shell structures	- It requires seed particles - Increase in complexity with multistep processes	[10]
	Miniemulsion polymerization	- Ability to encapsulate hydrophobic or hydrophilic substances (in direct or inverse miniemulsions, respectively)	- Need for high-shear forces (higher energy input)	[11,12,13,14,15]
	Microemulsion polymerization	- Very small particles (<100 nm) with narrow size distribution - Stable emulsions	- Low solid content - High surfactant concentration required	[16,17]
	Pickering emulsions	- Stabilized by solid particles instead of surfactants - Enhanced stability against coalescence - Ability to encapsulate substances	- Need for suitable solid particles for stabilization; limited to particles that can adsorb at the interface - Control of particle size can be complex	[18,19,20]
	Suspension polymerization	- No surfactant required	- Large polymer beads (in general, not suited for nanomaterials) - Broad particle size distribution - Need for mechanical stirring through the whole process	[21,22,23,24]
	Precipitation polymerization	- Simple setup	- Limited control of particle morphology	[25,26]
	Dispersion polymerization	- Quite uniform and spherical particles	- Limited to certain monomer/solvent combinations - Mostly suitable for submicron-sized particles	[27,28,29,30]
Without polymerization	Ouzo effect (spontaneous emulsification)	- Simple and fast - No need for surfactants or high-energy input - Suitable for already available polymers	- Limited to certain polymer–solvent combinations - Particle size control can be challenging	[31,32,33,34]
	Emulsion–solvent evaporation	- Good control over particle size - Suitable for already available polymers	- Need for volatile organic solvents - Residual solvent can be an issue - Stability of emulsions can be problematic	[35,36,37]
	Spraying techniques (spray drying or freezing)	- Scalable and suitable for large-scale production - Versatile for various polymers and compounds	- Requires specialized equipment - Broad particle size distribution - Energy-intensive processes	[38,39,40]
	Supercritical fluid techniques (SAS/RESS)	- Small and uniform particles - Environmentally friendly (solvent-free products)	- Requires high-pressure equipment - Limited to polymers soluble in supercritical fluids - High operational costs	[41,42,43]

## Data Availability

All data are available.
